# Global Action for Local Impact: The 11th International Conference on Typhoid and Other Invasive Salmonelloses

**DOI:** 10.1093/cid/ciaa236

**Published:** 2020-07-29

**Authors:** Noah Duff, A Duncan Steele, Denise Garrett

**Affiliations:** 1 Coalition against Typhoid, Sabin Vaccine Institute, Washington, USA; 2 Enteric and Diarrheal Diseases, Global Health, Bill & Melinda Gates Foundation, Seattle, Washington, USA

**Keywords:** enteric fever, typhoid, *Salmonella* Typhi, *Salmonella* Paratyphi, invasive nontyphoidal *Salmonella* disease

## Abstract

Typhoid and other invasive salmonelloses continue to cause an estimated 14.8 million cases and > 200 000 deaths annually, largely affecting children in low- and middle-income countries. However, recent strides in global policy have paved the way for accelerated progress with prevention and control efforts. To translate these recent advancements at the global level into real impact in communities at the local level, the Coalition against Typhoid, based at the Sabin Vaccine Institute, convened the 11th International Conference on Typhoid and Other Invasive Salmonelloses in Hanoi, Vietnam, in March 2019. Here, we review the significant topics and research discussed at the conference, including diagnostics, environmental surveillance, drug resistance, burden of disease, and vaccines, as well as additional prevention and control interventions.

Typhoid and paratyphoid fevers, collectively referred to as enteric fever, are bacterial infections in humans caused by, respectively, the bacterium *Salmonella enterica* subspecies *enterica* serovars Typhi (*S.* Typhi) and Paratyphi (*S.* Paratyphi) A, B, or C. These bacterial strains, combined with invasive nontyphoidal *Salmonella* (iNTS) serovars—including serovars Typhimurium and Enteritidis— result in a significant burden of disease, including morbidity, mortality, and financial cost, in many low- and middle-income countries lacking access to clean water and improved sanitation infrastructure. It is estimated that 14.3 million cases of typhoid and paratyphoid fevers occur globally each year, resulting in nearly 136 000 deaths annually; of these cases, > 76% are caused by *S.* Typhi alone [1]. However, iNTS disease, which causes an estimated 535 000 cases and > 77 000 deaths annually, is becoming an increasingly recognized threat in sub-Saharan Africa, where high rates of human immunodeficiency virus infections, malaria, and low sociodemographic development leave many, especially young children, vulnerable to the disease [2].

As part of the response to the high burden of enteric fever and iNTS disease, over the last few years there has been an unprecedented period of progress and coordination in the global public health prevention and control efforts for these diseases. For typhoid fever, the progress of the last 2 years has been markedly notable. In 2017, the World Health Organization (WHO) recommended and prequalified the first typhoid conjugate vaccine (TCV), Typbar-TCV (Bharat Biotech International Ltd, Hyderabad, India), for use in children > 6 months of age in endemic settings [3, 4]. Shortly thereafter, Gavi, the Vaccine Alliance (Gavi) announced US$85 million in funding to support TCV introduction in eligible countries, marking the first time a typhoid vaccine was eligible for Gavi funding [5]. Already, we are seeing the benefits of these global policies, as Pakistan recently became the first country in the world to introduce TCV into its routine immunization program in November 2019 [6], and both Zimbabwe and Liberia are preparing for their own Gavi-supported introductions in 2020–2021 [7].

Despite this progress, challenges remain. New vaccine introduction requires a high degree of involvement and coordination from government partners, who must contend with competing national priorities. There is no currently available vaccine for either paratyphoid fever or iNTS disease. Because of limitations of current diagnostic tools, diagnosis in most low-resource settings remains difficult, hampering policy decisions by ministries of health. The increasing incidence of multidrug-resistant bacterial *Salmonella* strains threatens our ability to treat these infections with threatening predictions of pre–antibiotic era mortality rates [8]. Climate change and rapid urbanization are altering the frequency of disease transmission through water scarcity and inadequate sanitation.

To address these challenges and sustain the momentum of the last 2 years, the Coalition against Typhoid at the Sabin Vaccine Institute convened the 11th International Conference on Typhoid and Other Invasive Salmonelloses in Hanoi, Vietnam, on 26–28 March 2019. This 3-day event, supported by the Bill & Melinda Gates Foundation, gathered > 400 experts from 46 countries—the largest event to date in the conference series’ 11-year history—to share breakthrough research and discuss innovative solutions to translate the recent global advancements in prevention and control efforts into real impact in the endemic communities most vulnerable to enteric fever and iNTS disease. With the introduction of new concurrent abstract sessions and interactive workshops, the conference showcased more data than ever before, with > 250 presentations covering a breadth of topics, such as burden of disease; environmental surveillance; novel diagnostics; vaccine development; immunology; genomics; new vaccine introduction; antimicrobial resistance; health economics; and advocacy and policy.

Here, we focus on several of the major themes discussed at the conference—including diagnostics, environmental surveillance, drug resistance, burden of disease, and vaccines, as well as additional prevention and control interventions—providing an overview on the progress and challenges related to each topic, which will be further described in the subsequent articles included in this supplement to *Clinical Infectious Diseases*.

## NOVEL TOOLS FOR DETECTION OF ENTERIC FEVER

Accurate diagnoses are imperative for ensuring that those who are sick receive timely medical attention and appropriate treatment. However, enteric fever and iNTS disease are notoriously difficult to diagnose, given the challenging nature of salmonellae organisms and associated symptomatic infections, which often resemble other infectious febrile illnesses, such as malaria or dengue. As a result, misdiagnoses and missed cases are commonplace, which could lead to the underestimation of current disease burden estimates for both enteric fever and iNTS disease. The current gold standard diagnostics for enteric fever rely on either blood culture, which is about 50% as sensitive as bone marrow culture, and which is expensive and resource intensive, and thus less than ideal for use in many low-resource, high-burden countries. In these settings, the more common diagnostic tool is the serological Widal test, which—while simple and inexpensive—was developed > 100 years ago and is frequently inaccurate due to cross-reactivity with other infectious agents. Several new diagnostic tools that aim to be cost-effective and rapidly deliver accurate results have been in development for years, and many of the discussions at the conference urged increased investment and a renewed, concerted effort to bring these new tools to market. In this supplement, Darton et al elaborate on this discussion, detailing these new tools and several approaches to utilizing improved diagnostics for enteric fever detection.

In addition to novel diagnostics, another promising tool for enteric fever detection that emerged at the conference was environmental surveillance. Environmental surveillance, which measures the abundance of *Salmonella* pathogens in the environment, could be an alternative, cost-efficient method to clinical surveillance in low-resource, high-burden settings. This type of surveillance can be helpful in determining transmission pathways and identifying risk factors within populations. In this supplement, Andrews et al review emerging approaches for environmental surveillance and address some of the associated challenges. Methods of environmental surveillance are further explored by Matrajt et al, while Li et al review advancements in developing new rapid monitoring platform technologies for bacterial detection in environmental samples at the point of collection.

## THE RISE OF DRUG RESISTANCE

Drug-resistant cases of enteric fever and iNTS disease are increasing globally, with several outbreaks occurring in 2019 alone, notably in highly endemic countries such as Pakistan and Zimbabwe. The rise of drug resistance threatens to derail much of the progress that has been made over the last 2 years, and increases the urgency with which the enteric fever community should address this growing issue—a rallying cry heard across several presentations at the conference. While drug resistance may help to strengthen the case for introducing TCV in areas with drug-resistant *S.* Typhi transmission, several presentations highlighted that drug-resistant profiles vary across pathogens and that drug-resistant plasmid transfer can occur between serovars [9], meaning that without an effective vaccine for paratyphoid fever and iNTS disease, drug-resistant cases of these illnesses could become untreatable. In this supplement, Saha et al delve deeper into these discussions and the data presented on drug resistance at the conference. Also included in this supplement are data from a case study during a drug-resistant outbreak in Blantyre, Malawi, by Olgemoweller et al, which demonstrate the severe consequences—including intestinal perforation and mortality—of managing a disease that is both unpreventable and untreatable.

## MEASURING THE GLOBAL BURDEN OF ENTERIC FEVER

Clinical surveillance data help to inform both accurate, comprehensive estimates on global burden of disease as well as decision making on the implementation and introduction of new control and prevention strategies. Over the last decade, we have seen the body of population-based surveillance data from endemic regions, including sub-Saharan Africa and South Asia, grow, helping to improve our understanding of the global incidence of enteric fever. This new data has inspired changes in global policy, including the 2018 WHO revised position paper, and has helped to make TCV introduction a priority for the most high-burden countries. The 2019 conference in Hanoi included presentations from many of these studies, including Surveillance of Enteric Fever in India (SEFI), the Surveillance for Enteric Fever in Asia Project (SEAP), the Severe Typhoid Fever Surveillance in Africa (SETA) program, and the Strategic Typhoid Alliance across Asia and Africa (STRATAA). The methodologies of these 4 landmark studies are reviewed in this supplement by Carey et al, highlighting the strengths and limitations of each study and helping to improve our understanding of the data they are generating. Similarly, Mejia et al delve one step further into the methodologies from both SEAP and SETA in measuring the high economic burden of enteric fever to both the Ministry of Health and to families, which has been one of the strongest arguments made in advocating for increased investment in control and prevention strategies. However, typhoid fever is not exclusive to sub-Saharan Africa and South Asia, but is also found in many Polynesian island nations across Oceania, including Samoa. Through > 10 years of surveillance data, Sikorski et al demonstrate the high incidence of typhoid fever in Samoa, which, in 2018, initiated the Samoa Typhoid Fever Control Program with a goal to immunize all Samoans, aged 1–49 years, with TCV.

While much of the focus in surveillance research and global policy over the last decade has concentrated on enteric fever, especially typhoid fever, iNTS disease is now emerging as a major public health priority, particularly in sub-Saharan Africa, where case fatality rates for iNTS disease often exceed that of enteric fever. With > 30 presentations and posters dedicated to the disease at the 2019 conference, presenters aimed to highlight the growing importance of this disease. However, data gaps remain, impeding further advancements in control and prevention strategies. In this supplement, Kariuki and Ouwuso-Dabo address several of these gaps in their overview of the iNTS disease presentations and discussions from the conference, where they argue for a renewed global effort over the next few years to improve the collective understanding of this fatal disease. Renewed efforts will build on the current surveillance data that exist, such as the 15 years of surveillance data from a national pediatric hospital in Bamako, Mali, that was presented by William Still of the University of Maryland School of Medicine, which demonstrated the high burden of iNTS disease in that country and pointed to the opportunity for a novel vaccine candidate that would protect against the 4 most common iNTS serovars: *S.* Enteritidis, *S.* Typhimurium, *Salmonella* Dublin, and I:4. The complete results and analyses from these surveillance data are detailed in this supplement by Still et al.

## PREVENTIVE INTERVENTIONS FOR ENTERIC FEVER

Transmission of enteric fever and iNTS disease is directly linked to poor water, sanitation, and hygiene (WASH) infrastructure and, as such, safe food, clean water, improved sanitation, and good hygiene practices are key to preventing these diseases. However, improving WASH infrastructure requires a considerable financial investment and can take years to implement—years that we likely do not have as increasing rates of drug resistance render current treatment options for enteric fever and iNTS disease ineffective. As such, our best guarantee for prevention and control of these diseases in the near term is vaccines. While vaccines for typhoid fever have existed for decades, they have not been widely used in routine immunization programs given significant limitations, such as reduced effectiveness and duration of protection. However, over the last 2 years a new WHO-prequalified and -recommended TCV has become commercially available in India and accessible in Gavi-eligible, high-burden countries for introduction into routine immunization programs—a notable feat that was thoroughly discussed at the conference. While this is the first licensed TCV, several others are currently in the development pipeline, which Sushant Sahastrabuddhe of the International Vaccine Institute described in the conference’s opening plenary session. In this supplement, Sahastrabuddhe et al expand on this presentation in the review of the current typhoid vaccine candidates under development, as well as some of the challenges ahead for new vaccine introduction.

Following the excitement and momentum surrounding the introduction of new vaccines for typhoid fever, one of the major calls to action to emerge from the 2019 conference was the increased investment in developing vaccines against paratyphoid fever and iNTS disease. The growing body of data on paratyphoid fever and iNTS disease presented at the conference highlighted this unmet need and the potential impact of introducing such vaccines, particularly in sub-Saharan Africa for iNTS disease. Scott Baliban of the University of Maryland School of Medicine and Richard Malley of Boston Children’s Hospital each presented on promising new candidates and technologies for both diseases during the conference; they include these early-stage clinical candidates and others in their joint supplement overview article of the current iNTS disease and paratyphoid fever vaccine development landscape, highlighting challenges and several directions for continued research and development of these candidates in the ensuing years.

## TYPHOID CONJUGATE VACCINES AND THE FUTURE OF ENTERIC FEVER CONTROL

### Typhoid Conjugate Vaccines

Much of the 2019 conference was framed in the context of the introduction of new TCVs—the catalyst for the staggering progress made over the last 2 years, and one of the leading priorities for the enteric fever community in the immediate years to come. This progress has largely been driven and coordinated by the Typhoid Vaccine Acceleration Consortium (TyVAC), which was launched in 2017 to accelerate the introduction of TCVs in low-resource countries. Data from several of the TyVAC studies were presented at the conference, including data on safety and immunogenicity from Bangladesh, Malawi, and Nepal, as well as interim efficacy data from Nepal, which was recently published and showed TCV to be > 81% effective in protecting study participants from typhoid fever [10]. In this supplement, Neuzil et al summarize the TyVAC data presented at the conference.

Another contributor to the recent success with TCV has been Gavi, which in 2008 made the decision to support TCV candidates as they became licensed and in 2017—following the recommendation of Strategic Advisory Group of Experts on Immunization, and pending WHO prequalification of the first licensed TCV, Typbar-TCV—opened a funding window to support TCV introduction. Through this support, several countries, including Pakistan, Zimbabwe, and Liberia, have already been approved for Gavi-supported introduction of TCV in 2019–2020. The history of Gavi’s support for TCV, TCV application criteria, and initial lessons learned for investment decision making and new vaccine implementation are detailed in the Gavi supplement position piece by Soble et al.

However, national introduction of TCV may not be possible for every endemic country, given competing priorities for national policymakers, a paucity of convincing national data for decision makers, and Gavi eligibility criteria. As such, alternative vaccination strategies, such as the subnational vaccination strategies described in this supplement by Muhib et al, may prove to be a useful solution for national decision makers while also ensuring the targeted reduction of typhoid transmission in the most vulnerable communities. One such approach in the context of Navi Mumbai, India, was presented at the conference by Kashmira Date of the US Centers for Disease Control and Prevention; the complete results and lessons learned from this first public-sector, municipal-level introduction of TCV in India are outlined in this supplement.

### The Future of Enteric Fever Control

In light of the recent developments and promise of TCV introduction, many of the forward-thinking conversations at the 2019 conference circled around the idea of elimination—an idea once thought to be long out of reach. The closing plenary session at the conference, “Imagine: Global Actions for Local Impact Toward Elimination,” was entirely devoted to the topic. Presenters—including Jeffrey Stanaway of the University of Washington’s Institute for Health Metrics and Evaluation; Phionah Atuhebwe of WHO’s Regional Office for Africa; Stephen Luby of Stanford University; and John Crump of the University of Otago—all hypothesized on what elimination of typhoid fever could look like, how TCV implementation could get us there, and what obstacles must first be overcome. What became evidently clear from these presentations was that while widespread TCV introduction should be pursued as a means to quickly reduce burden, elimination of typhoid fever will likely take decades, given the likelihood of “shocks” over the ensuing years, such as climate change and rapid urbanization. The presenters come together in Stanaway et al’s article in this supplement to review and discuss several considerations for typhoid fever elimination. These considerations will likely be influenced over the coming years as TCV continues to be introduced and implemented. As Steele et al of the Bill & Melinda Gates Foundation urge in this supplement, the enteric fever community must remain vigilant with TCV introduction, ensuring that the benefits of TCVs reach those most at risk.

## CONCLUSIONS

The 11th International Conference on Typhoid and Other Invasive Salmonelloses provided an opportunity to both celebrate the recent advances and progress in enteric fever and iNTS disease control and prevention, as well as to acknowledge and identify the challenges that must be overcome to further these efforts and to ensure that enteric fever and iNTS disease become public health nuisances of the past. To get there, the global public health community has much work to accomplish. We should heed the calls to action that were heard in Hanoi—and discussed in the subsequent articles in this supplement—including renewed and concerted efforts to develop new diagnostics, addressing drug resistance, investing in paratyphoid fever and iNTS disease research, and engaging with the WASH community. We must build on our recent successes, using them to catalyze us to greater progress and innovation over the next 2 years. The results of this progress, and the strategies and approaches that contributed, will be discussed at the 12th International Conference on Typhoid and Other Invasive Salmonelloses in Cape Town, South Africa, 16–18 March 2021.

## Notes

***Financial support.*** This work was supported by the Bill & Melinda Gates Foundation (grant number OPP1182032).

***Supplement*** ***sponsorship.*** This supplement is funded with support from the Coalition against Typhoid Secretariat, housed at the Sabin Vaccine Institute in Washington, DC and made possible by a grant from the Bill & Melinda Gates Foundation.

***Potential conflicts of interest.*** The authors: No reported conflicts of interest. All authors have submitted the ICMJE Form for Disclosure of Potential Conflicts of Interest.
